# Topical Administration of a Mixed Microbial Culture of *Lactobacillus paracasei*, *Pichia membranifaciens* and *Saccharomyces cerevisiae* Significantly Inhibits the Development of Atopic Dermatitis in a Mouse Model Through IL-10 Overexpression by Dendritic Cells

**DOI:** 10.3390/biomedicines13102536

**Published:** 2025-10-17

**Authors:** Mao Kaneki, Chiharu Ohira, Tensei Magami, Aika Hamauzu, Yukari Inaba, Hideo Togase, Tomoki Fukuyama

**Affiliations:** 1 Laboratory of Veterinary Pharmacology, School of Veterinary Medicine, Azabu University, 1-17-71 Fuchinobe, Chuo-ku, Sagamihara-shi 252-5201, Kanagawa, Japan; da2301@azabu-u.ac.jp (M.K.); da2302@azabu-u.ac.jp (C.O.);; 2 Litanial Bioscience Laboratory, 2-26 Shinobe-Kitamachi, Befu-cho, Kakogawa 675-0121, Hyogo, Japan; 3 Center for Human and Animal Symbiosis Science, Azabu University, 1-17-71 Fuchinobe, Chuo-ku, Sagamihara-shi 252-5201, Kanagawa, Japan

**Keywords:** *Lactobacillus paracasei*, *Saccharomyces cereviciae*, *Pichia membranifaciens*, atopic dermatitis, dendritic cells, IL-10

## Abstract

**Background/Objectives**: In this study, we focused on a mixed microbial culture of *Lactobacillus paracasei*, *Pichia membranifaciens*, and *Saccharomyces cerevisiae* (LS) as a new probiotic and examined the therapeutic and preventive effects of topical treatment with LS in a mouse model of atopic dermatitis (AD). **Methods**: Immunomodulatory effects of LS were examined with murine dendritic cell lines (DC2.4) by measuring the interleukin (IL)-10 and tumor necrosis factor (TNF) α levels. The anti-inflammatory effects of LS were evaluated in stimulated human epidermal keratinocytes (HaCaTs) by focusing on the production of IL-8 and thymus and activation-regulated chemokine (TARC). Therapeutic and preventive properties of topical treatment with LS (10%) were finally examined in a mouse model of AD developed by topical sensitization to house dust mite ointment. Clinical symptoms, back skin thickness, and transepidermal water loss (TEWL) were monitored weekly, and the immune responses in the auricular lymph nodes were analyzed after necropsy. **Results**: LS treatment significantly enhanced the secretions of IL-10 and TNFα by DC2.4 cells. IL-8 and TARC production by stimulated HaCaT cells was significantly decreased by co-culturing with LS. Although there were no significant changes in clinical symptoms, skin thickness, or TEWL in the therapeutic setting of the AD mouse model, the number of IgE-positive B cells and IL-4 levels in the local lymph nodes significantly decreased in the LS treatment group. Preventive treatment with LS significantly decreased AD symptoms compared to those in AD control mice. **Conclusions**: Our findings indicate that the immunomodulatory and anti-inflammatory effects of LS prevent the development of AD.

## 1. Introduction

The close relationship between the skin microbiota and the aggravation or amelioration of atopic dermatitis (AD) has been well documented [1,2]. Consequently, probiotics have long been investigated as alternative strategies for preventing and alleviating AD symptoms in humans and companion animals, with oral administration being the most common route [3,4,5]. *Lactobacillus paracasei* (*L. paracasei*) is a representative probiotic that has shown efficacy against several allergic diseases, including AD, atopic eczema, asthma, and allergic rhinitis [6,7,8,9]. In addition to its barrier-supporting functions, *L. paracasei* acts as an immune modulator; oral administration has demonstrated immunomodulatory effects in vivo and in vitro [10,11,12]. Dendritic cells (DCs) are major targets of *L. paracasei* and play a central role in antigen uptake and presentation during allergic responses [13]. For example, Mileti et al. [14] reported that *L. paracasei* not only induced minimal cytokine release but also suppressed DC-driven production of inflammatory cytokines such as interleukin (IL)-12 and tumor necrosis factor (TNF)-α, thereby limiting Th1 polarization in response to Salmonella. Although promising, the immunomodulatory effects of *L. paracasei* alone are often modest, suggesting the need for complementary microbial partners to enhance efficacy.

The skin, beyond being a physical barrier, is a complex immunological organ colonized by a diverse community of bacteria, fungi, and viruses that actively contribute to immune homeostasis [15]. Commensal microbes stimulate antimicrobial peptide production in keratinocytes, educate resident innate immune cells, and help restrict pathogen expansion, such as *Staphylococcus aureus* (*S. aureus*) suppression by commensal coagulase-negative staphylococci [16]. Dysbiosis in AD, characterized by the loss of beneficial commensals and the overgrowth of *S. aureus*, is closely linked to barrier dysfunction and exaggerated type 2 immunity [16]. Because many of the key pathogenic events occur locally—through keratinocyte-derived alarmins, dendritic cell activation, and tissue-resident T cell responses—topical application of probiotics or their metabolites represents a rational strategy to restore immune balance at the site of disease. Compared with oral delivery, the dermal route offers the advantage of acting directly on keratinocytes, skin-resident DCs, and the local microbiota to (i) reduce pathogen colonization, (ii) enhance antimicrobial peptide expression and barrier function, and (iii) reprogram antigen presentation toward regulatory rather than inflammatory responses, all while minimizing systemic exposure.

Several clinical and preclinical studies support the potential of topical probiotic interventions. Formulations containing non-pathogenic bacterial lysates or heat-treated probiotics, such as *Vitreoscilla filiformis* lysate and heat-treated *Lactobacillus johnsonii* NCC 533, have been shown to reduce *S. aureus* colonization, improve clinical scores, and enhance skin barrier function. In one exploratory study, a lotion containing heat-treated *L. johnsonii* lowered *S. aureus* load in AD patients and correlated with clinical improvement, while a placebo-controlled trial of *V. filiformis* lysate demonstrated reductions in transepidermal water loss (TEWL) [17,18,19]. More recently, first-in-human studies using topical transfer of the commensal Roseomonas mucosa confirmed safety and decreased disease severity as well as topical steroid requirements [20]. Systematic reviews of topical probiotics for AD underscore both the promise and heterogeneity of outcomes, emphasizing the need for strain-specific mechanistic studies [21].

Based on this rationale, we developed a mixed microbial culture of *L. paracasei*, *Pichia membranifaciens* (*P. membranifaciens*), and *Saccharomyces cerevisiae* (*S. cerevisiae*)—referred to as LS—as a topical probiotic candidate for AD. *L. paracasei* was selected for its immunomodulatory activity, while *P. membranifaciens* was included for its ability to secrete bioactive proteins and metabolites that may inhibit pathogenic competitors and stabilize microbial communities. *S. cerevisiae* contributes β-glucans, mannans, and secreted metabolites that modulate innate immune responses and support bacterial survival [22,23]. Prior evidence of synergistic protective effects of this mixed culture in other biological systems (e.g., aquaculture) further justified its use. Together, these complementary properties suggest that LS could confer combined immune-modulatory, barrier-supporting, and ecological-stabilizing effects on the skin [16,24].

We hypothesize that topical LS exerts benefit through three, non-exclusive mechanisms: (1) direct immunomodulation of skin antigen-presenting cells—specifically induction of regulatory cytokines (IL-10) from dendritic cells and promotion of Treg/bystander suppression that dampen Th2 polarization; (2) suppression of inflammatory signaling in keratinocytes (reduced IL-8/TARC), helping to lower recruitment of inflammatory leukocytes and reduce alarmin production; and (3) ecological modulation of the local microbiota (competition or niche modulation that reduces pro-inflammatory organisms such as S. aureus and promotes antimicrobial peptide expression). These mechanisms predict stronger preventive effects (when barrier and microbial niches are not yet extensively disrupted) and more modest effects when applied after chronic lesions and established dysbiosis. The experiments below test signatures of (1) dendritic cell IL-10/TNFα induction, (2) keratinocyte cytokine suppression, (3) local LN Th2 markers (IL-4/IL-13) and B-cell IgE, and (4) histological readouts of barrier integrity.

The objective of this study was to evaluate both the preventive and therapeutic effects of dermally applied LS in a murine model of AD. In addition, we investigated its immunomodulatory properties using dendritic cells and keratinocytes in vitro, with the goal of elucidating potential mechanisms of action relevant to skin immune homeostasis.

## 2. Materials and Methods

The LS culture was provided by Litanial Bio Science, Co., Ltd. (Hyogo, Japan) [25]. This culture was prepared by co-cultivation of *L. paracasei*, *P. membranifaciens*, and *S. cerevisiae* in a rice grain broth supplemented with 5% dextrose at 30 °C for 24 h. Sterility was assessed by cultivating the microbial mixture on heart infusion (HI) agar (Nissui Pharmaceutical Co., Ltd., Tokyo, Japan). The immunomodulatory and anti-inflammatory effects of LS were examined in vitro using murine dendritic cell lines (DC2.4) and a human epidermal keratinocyte cell line (HaCaT). DC2.4 was obtained from the American Type Culture Collection (Manassas, VA, USA) and was cultured in RPMI 1640 medium (FUJIFILM Wako Pure Chemical Corporation, Osaka, Japan) supplemented with 10% fetal calf serum (FCS; Sigma-Aldrich Co., LLC., Tokyo, Japan) and penicillin–streptomycin (FUJIFILM Wako Pure Chemical Corporation). HaCaT cells were obtained from CLS (Cell Lines Service) GmbH (Eppelheim, Germany) and cultured in Dulbecco’s modified Eagle’s medium (FUJIFILM Wako Pure Chemical Corporation) supplemented with 10% FCS and penicillin–streptomycin. DC2.4 cells (1 × 10^4^ cells/100 μL) at 70% confluency were seeded in a 96-well culture plate and exposed to several concentrations of LS (0, 0.125, 0.25, 0.5, and 1 mg/mL). The concentrations of IL-10 and TNFα in the supernatants were quantified by enzyme-linked immunosorbent assay (ELISA) (DuoSet ELISA kit, R&D Systems, Minneapolis, MN, USA). HaCaT cells (1 × 10^4^ cells) were seeded in 100 μL of the medium at 70% confluence in 96-well culture plates and stimulated with recombinant human TNFα and interferon (IFN) γ (PeproTech, Inc., Cranbury, NJ) for 1 h; next, 100 μL of several concentrations of LS (0, 0.125, 0.25, 0.5, and 1 mg/mL) in culture medium was added, followed by incubation for 24 h. The concentrations of IL-8 and thymus activation-regulated chemokine (TARC) in the supernatants were quantified by ELISA. The therapeutic and preventive properties of topical LS treatment were examined in a mouse model of AD. The AD mouse model was generated by topical sensitization with Biostir AD (*Dermatophagoides farinae* extract) in addition to the topical application of 4% sodium dodecyl sulfate solution (FUJIFILM Wako Pure Chemical Corporation) in NC/Nga mice according to a previous report [26]. The treatment regimen involved once daily topical application (0.1 mL/mouse) of 10% of LS solution (*n* = 8) or DW vehicle (*n* = 7). Treatment was initiated on day 19 after the development of AD, when the mean AD score was 2.07. Preventive treatment was administered from day 0 to day 18, prior to AD induction, whereas therapeutic treatment was initiated after lesion onset (day 19 to day 33; mean AD score at initiation = 2.07). TEWL, back skin thicknesses, and clinical scores were monitored once weekly during the experimental period. TEWL was measured using a VAPO SCAN (AS-VT100RS, ASCH JAPAN Co., LTD, Tokyo, Japan), and a clinical score of 0–4 was assigned as follows: no symptoms, 0; mild, 1; moderate, 2; severe, 3; and extreme, 4 for the ear and back, as previously described [27]. Auricular lymph node (LN) samples were collected from each mouse 1 d after the final sensitization. Single-cell suspensions isolated from the LN were prepared as described previously [28,29], and the total number of cells was counted using a CellDrop™ Cell Counting System (DeNovix Inc., Wilmington, DE, USA). The cells were analyzed using a BD FACSAria™ III cell sorter (BD Biosciences, Tokyo, Japan) with monoclonal antibodies (PE/Cyanine7-conjugated anti-mouse CD3, PE-conjugated anti-mouse CD4, PE-conjugated anti-mouse CD11b, APC-conjugated anti-mouse CD11c, PerCP/Cyanine5.5-conjugated anti-mouse CD19, APC-conjugated anti-mouse CD44, APC/Cyanine7-conjugated anti-mouse CD62L, FITC-conjugated anti-mouse IgE, FITC-conjugated anti-mouse MHC class II, and DAPI) (BioLegend Inc., San Diego, CA, USA; Miltenyi Biotec K.K. and Sony Biotechnology Inc., Tokyo, Japan). Single-cell suspensions of LNs were used to evaluate cytokine release by T cells. Single-cell suspensions of LNs (5 × 10^5^ cells/well) were incubated with mouse T-activator CD3/CD28 dynabeads (Thermo Fisher Scientific Inc., Yokohama, Kanagawa, Japan) for 24 h. The levels of interferon (IFN) γ, IL-4, IL-13, and IL-17 in the supernatant were evaluated using ELISA (DuoSet ELISA kit, R&D Systems). Semi-quantitative histopathological evaluation of a portion of the skin sample was performed in a blinded fashion using the following grading system: 0, within normal limits; 1, mild; 2, moderate; 3, severe. The total lesion score was used for the statistical evaluation. Data are expressed as mean ± 1 standard error of the mean (SEM). Analysis of variance (ANOVA) followed by Dunnett’s multiple-comparison test was used to evaluate the results of the in vitro studies. In in vivo experiments, 2-way ANOVA followed by Šídák’s multiple-comparison test or Student’s *t*-test was used to test the significance of differences between the two groups. Statistical significance was estimated at 5% levels of probability, and data were analyzed using GraphPad Prism 10 (GraphPad Software, San Diego, CA, USA).

## 3. Results

**Figure 1:**
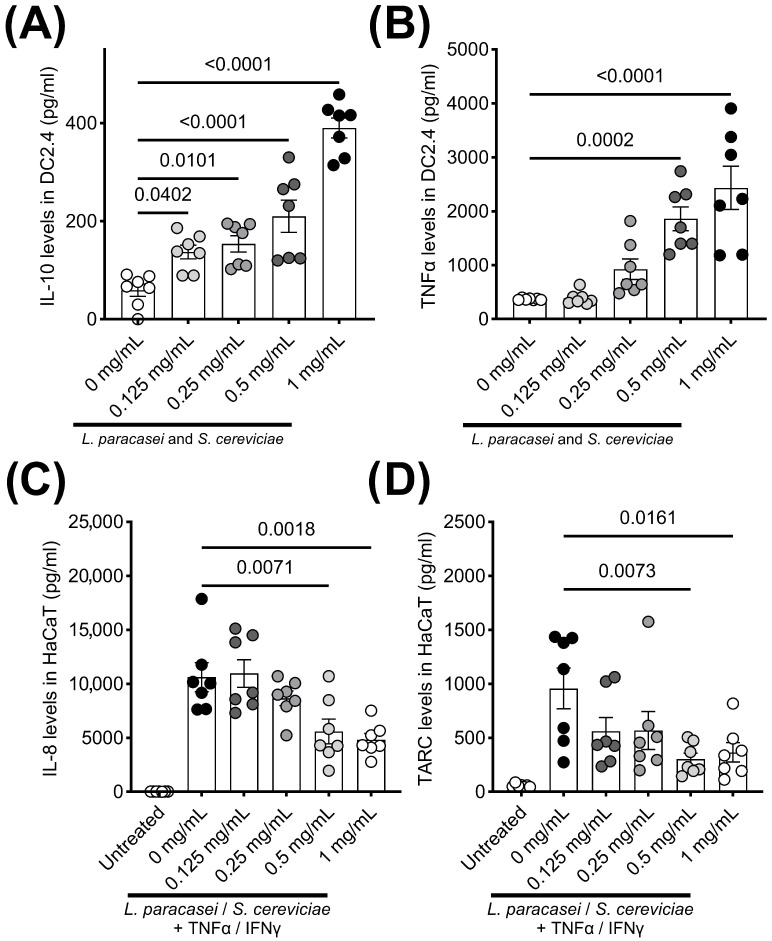
Influence of LS treatment on cytokine secretion by dendritic cells and epidermal keratinocytes. LS treatment significantly enhanced the production of (**A**) IL-10 and (**B**) TNFα in the murine dendritic cell line (DC2.4). In contrast, (**C**) IL-8 and (**D**) TARC secretion by TNFα/IFNγ-stimulated human epidermal keratinocyte cell line (HaCaT) were significantly inhibited by LS treatment in a dose-dependent manner. Each result is presented as the mean (pg/mL) ± 1 SEM. *n* = 7 per group. *p* < 0.05 (Dunnett’s multiple-comparison test) vs. the control group. IFN, interferon; IL, interleukin; *L. paracasei*, *Lactobacillus paracasei*; *S. cerevisiae*, *Saccharomyces cerevisiae*; TARC, thymus activation-regulated chemokine; TNF, tumor necrosis factor; “+” means that TNF/IFN was treated in addition to bacteria.

**Figure 2:**
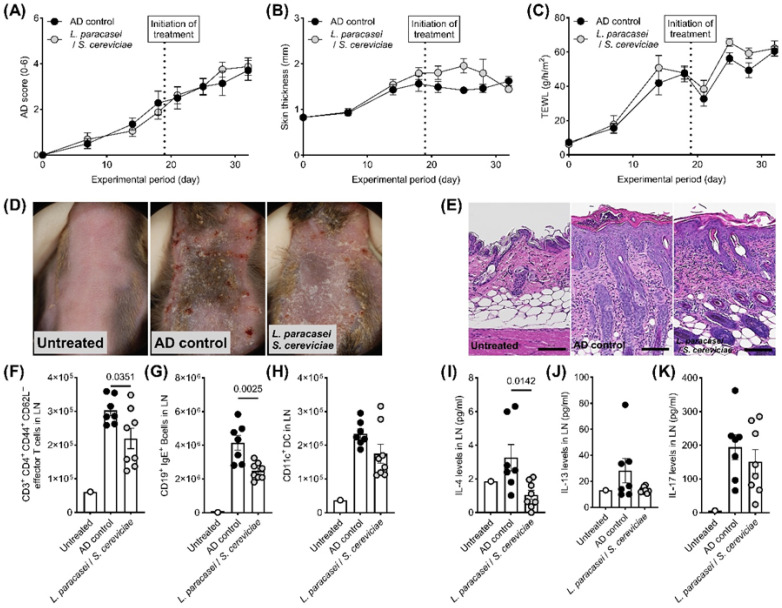
Effects of topical LS treatment in a therapeutic AD mouse model. In the therapeutic setting, topical LS treatment did not alter (**A**) AD clinical score, (**B**) skin thickness, or (**C**) TEWL compared with the AD control group. (**D**) Representative macroscopic images of skin lesions and (**E**) histological sections of affected skin (scale bar = 100 μm). LS application significantly reduced the number of (**F**) CD3^+^CD4^+^CD44^+^CD62L^−^ effector T cells and (**G**) CD19^+^IgE^+^ B cells in auricular lymph nodes, whereas (**H**) CD11c^+^ dendritic cell counts were unchanged. LS also significantly decreased (**I**) IL-4 levels, but did not affect (**J**) IL-13 or (**K**) IL-17 expression in auricular lymph nodes. Data are shown as mean ± standard error of the mean (SEM). Group sizes: untreated (*n* = 1), AD control (*n* = 6), LS treatment (*n* = 7). *p* < 0.05 (Student’s *t*-test) vs. AD control. AD, atopic dermatitis; TEWL, transepidermal water loss.

**Figure 3:**
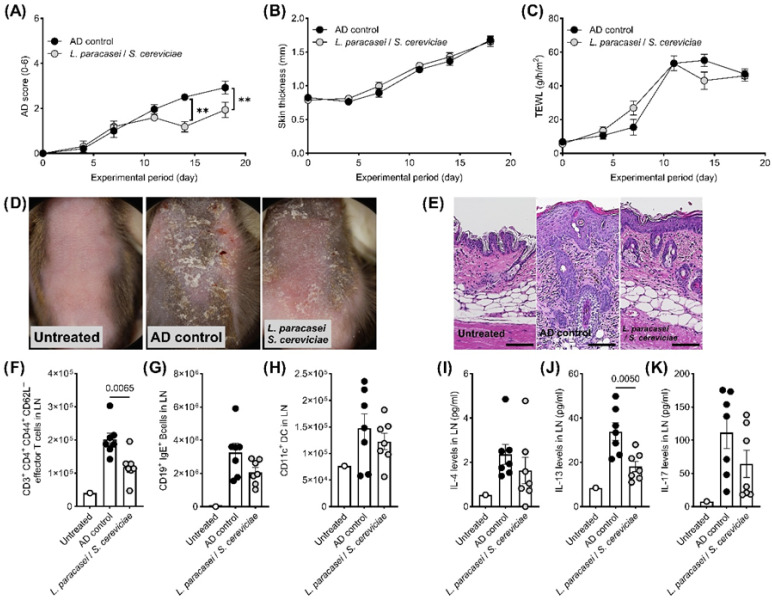
Preventive effects of topical LS treatment in an AD mouse model. In the preventive setting, topical LS treatment significantly reduced (**A**) AD clinical score, while (**B**) skin thickness and (**C**) TEWL remained unchanged compared with the AD control group. (**D**) Representative macroscopic images of skin lesions and (**E**) histological sections of affected skin (scale bar = 100 μm). LS application significantly decreased the number of (**F**) CD3^+^CD4^+^CD44^+^CD62L^−^ effector T cells in auricular lymph nodes, but had no effect on (**G**) CD19^+^IgE^+^ B cells or (**H**) CD11c^+^ dendritic cells. Among cytokines, (**I**) IL-4 levels were significantly reduced, whereas (**J**) IL-13 and (**K**) IL-17 levels were unaffected. Data are shown as mean ± standard error of the mean (SEM). Group sizes: untreated (*n* = 1), AD control (*n* = 6), LS treatment (*n* = 7). ** *p* < 0.01 (Student’s *t*-test) vs. AD control. AD, atopic dermatitis; TEWL, transepidermal water loss.

LS treatment significantly enhanced the secretions of IL-10 and TNFα by DC2.4 cells (Figure 1A,B). In contrast, IL-8 and TARC production by stimulated HaCaT cells was significantly decreased by co-culturing with LS (Figure 1C,D). Although there were no significant changes in clinical symptoms, skin thickness, and TEWL in the therapeutic setting of the AD mouse model (Figure 2A–D), histological evaluations, including hyperplasia in the keratinized layer and crust in the epidermis, were significantly ameliorated by LS treatment (Table 1, Figure 2E). Allergy-related immune reactions, including the number of IgE-positive B cells and IL-4 levels in the local lymph nodes, also significantly decreased in the LS treatment group (Figure 2F–K). The effects of LS were highlighted more in preventive treatment. LS treatment significantly decreased AD symptoms (Figure 3A,D) and histological findings (Table 1, Figure 3E), whereas skin thickness and TEWL were unaffected (Figure 3B,C). Effector T cells and IL-13 levels in the LN in the LS treatment group were significantly reduced compared to those in AD control mice (Figure 3F–K).

## 4. Discussion

In this study, we demonstrated that a mixed microbial culture of *L. paracasei*, *P. membranifaciens*, and *S. cerevisiae* (LS) exerted both preventive and therapeutic effects in a murine model of AD, with preventive application showing the strongest benefits. The superior preventive efficacy suggests that LS is most effective at modulating early immune priming and barrier function before chronic inflammation and dysbiosis are firmly established. Once lesions develop, structural barrier damage, altered lipid profiles, and dominant *S. aureus* colonization likely create a more resistant niche where topical probiotics alone cannot fully reverse disease, consistent with observations from both animal and human topical probiotic studies [16,21]. This distinction has implications for potential clinical application, suggesting LS may be better suited as a prophylactic or maintenance adjunct rather than a rescue treatment.

The rationale for including *L. paracasei*, *P. membranifaciens*, and *S. cerevisiae* rests on their complementary functions. Train-specific contributions may underlie the observed synergy. *L. paracasei* is known for its immunomodulatory activity, particularly induction of tolerogenic DCs and IL-10 production, which was recapitulated in our in vitro DC2.4 assays [11]. Yeast-derived components from *S. cerevisiae* (β-glucans, mannans) are potent activators of pattern recognition receptors and can enhance innate barrier defenses while attenuating keratinocyte-derived inflammatory signals such as IL-8 and TARC [24]. *P. membranifaciens* produces bioactive metabolites with antimicrobial activity, potentially suppressing opportunistic organisms and stabilizing the local microbial community, thereby creating a favorable niche for Lactobacillus persistence [23]. Together, these complementary roles likely contributed to reduced Th2 cytokines, IgE+ B cells, and epidermal inflammation in treated animals.

An intriguing finding of this study is the concurrent induction of IL-10 and TNF-α in DCs. While IL-10 is a hallmark anti-inflammatory cytokine, TNF-α is generally pro-inflammatory [30,31]. The co-induction suggests LS may not act through simple suppression but rather through immune rebalancing: promoting regulatory pathways (via IL-10) while also maintaining controlled inflammatory signals (via TNF-α) that support antimicrobial defense. Such a dual response may prevent excessive Th2 polarization without compromising host protection. Further investigation using primary human DCs and in vivo cytokine neutralization experiments will be needed to clarify this dual impact.

Although LS modulated key immune pathways—reducing IL-4, IL-8, and TARC levels—it did not achieve significant clinical improvements in the therapeutic setting. This gap between immunological modulation and overt disease resolution may reflect insufficient dosing, limited penetration of active components, or the difficulty of reversing established pathology. Optimizing the formulation, application frequency, or combining LS with barrier-repair strategies may improve therapeutic efficacy. Additionally, expanding cytokine profiling (IL-5, IL-13, and IFN-γ) and T cell subset analysis (Th2, Th17, and Tregs) would provide a more comprehensive picture of LS’s immunological footprint.

These findings align with emerging translational efforts in humans. Topical application of *Vitreoscilla filiformis* lysate and heat-treated *L. johnsonii* formulations has demonstrated improvements in TEWL and reductions in *S. aureus* colonization in AD patients, while live Roseomonas mucosa biotherapy recently showed safety and clinical benefit in children with AD [17,19,20]. Our results support the feasibility of live or mixed microbial topical approaches and highlight that prophylactic or maintenance applications may be especially valuable for high-risk individuals prone to recurrent flares.

It is important to emphasize that these results derive from murine and in vitro models, which do not fully replicate the human skin immune microenvironment. While promising, translation to humans or companion animals requires caution. Future studies should include safety assessments, allergenicity testing, and exploration of regulatory challenges associated with live microbial applications. Methodological details also require clarification for reproducibility and translational relevance. Factors such as strain viability, the exact composition of the topical formulation, and application parameters (dose, frequency, and duration) should be explicitly reported. These details will be critical for replication and for evaluating clinical feasibility.

In conclusion, topical application of a mixed culture of *L. paracasei*, *P. membranifaciens*, and *S. cerevisiae* (LS) demonstrated immunomodulatory and anti-inflammatory activity in vitro and in a murine AD model, with preventive effects more pronounced than therapeutic ones. These findings highlight the potential of early, localized microbiome modulation as a strategy to prevent or attenuate AD development. However, the study is limited by its reliance on murine and in vitro models, the absence of single-strain controls, and incomplete mechanistic characterization. Translational application will require further investigation into safety, formulation optimization, broader immunological profiling, and confirmation in human clinical trials. Future studies should focus on defining strain-specific contributions, clarifying the role of IL-10/TNF-α co-induction, and evaluating long-term effects in preventive versus therapeutic contexts.

## Figures and Tables

**Table 1:** Histological evaluation of the back skin of a mouse model of atopic dermatitis.

	Untreated (*n* = 1)	AD Control (*n* = 7)	*L. paracasei* and *S. cereviciae* (*n* = 8)
Therapeutic setting			
Epidermis			
Parakeratosis	0.00 ± 0.00	1.00 ± 0.00	0.75 ± 0.16
Hyperplasia in keratinized layer	0.00 ± 0.00	2.43 ± 0.20	1.75 ± 0.14 ^*p* = 0.0205^
Crust	0.00 ± 0.00	2.29 ± 0.29	0.75 ± 0.16 ^*p* = 0.0003^
Hyperplasia in non-keratinized layer	0.00 ± 0.00	2.14 ± 0.14	2.00 ± 0.00
Ulcer	0.00 ± 0.00	0.86 ± 0.34	0.50 ± 0.19
Dermis			
Inflammatory cell infiltration	0.00 ± 0.00	2.86 ± 0.14	2.13 ± 0.40
Preventive setting			
Epidermis			
Parakeratosis	0.00 ± 0.00	1.29 ± 0.18	0.63 ± 0.26
Hyperplasia in keratinized layer	0.00 ± 0.00	1.86 ± 0.26	0.88 ± 0.23 ^*p* = 0.0135^
Crust	0.00 ± 0.00	2.14 ± 0.26	0.63 ± 0.38 ^*p* = 0.0066^
Hyperplasia in non-keratinized layer	0.00 ± 0.00	2.00 ± 0.00	1.88 ± 0.13
Ulcer	0.00 ± 0.00	1.00 ± 0.31	0.25 ± 0.25
Dermis			
Inflammatory cell infiltration	0.00 ± 0.00	3.00 ± 0.00	1.00 ± 0.38 ^*p* = 0.0003^

A histological score (0, within normal limits; 1, mild; 2, moderate; 3, severe) was assigned to each observation. Results are expressed as mean ± SEM. *p* < 0.05 (unpaired *t*-test) compared to the AD control group. AD, atopic dermatitis; SEM, standard error of the mean.

## Data Availability

The original contributions of this study are included in this article. Further inquiries can be directed to the corresponding author.

## Abbreviations

The following abbreviations are used in this manuscript:

AD	Atopic dermatitis
ANOVA	Analysis of variance
DCs	Dendritic cells
FCS	Fetal calf serum
HaCaT	Human epidermal keratinocytes
IFN	Interferon
Ig	Immunoglobulin
IL	Interleukin
LN	Lymph nodes
LS	Mixed microbial culture of *Lactobacillus paracasei, Pichia membranifaciens*, and *Saccharomyces cerevisiae*
SEM	Standard error of the mean
TARC	Thymus and activation-regulated chemokine
TEWL	Transepidermal water loss
TNF	Tumor necrosis factor
